# Risk of stillbirth among pregnant women with anaemia in India: secondary analysis of multiple cohorts from the ICMR-SPIC consortium

**DOI:** 10.1016/j.lansea.2026.100825

**Published:** 2026-07-31

**Authors:** Prerna Gopal, Charles Opondo, Debarati Mukherjee, Ayushi, Rukman Manapurath, John Michael Raj, Ramachandran Thiruvengadam, Ranadip Chowdhury, Pratibha Dwarkanath, Nitya Wadhwa, Kalpana Basany, Giridhara Rathnaiah Babu, Saswati Sanyal Choudhury, Shivaprasad S. Goudar, Archana Patel, Chittaranjan S. Yajnik, Sadhana Joshi, Sunita Taneja, Reema Mukherjee, Manisha Nair, ICMR-SPIC consortium, Pratibha Dwarkanath, John Michael Raj, Anura V. Kurpad, Tinku Thomas, Rahul Jain, Nitya Wadhwa, Shinjini Bhatnagar, Kalpana Basany, Shailendra Dandge, Catherine L. Haggerty, Cleareann H. Bunker, P.S. Reddy, Giridhara R. Babu, Deepa R, Suganya Rajendran, Yamuna Ana, Manisha Nair, Saswati Sanyal Choudhury, Farzana Zahir, Carolin Solomi V, Ashok Verma, Sereesha Rao, Gitanjali Deka, Pranabika Mahanta, Swapna Kakoty, Robin Medhi, Shakuntala Chhabra, Anjali Rani, Amrit Bora, Indrani Roy, Bina Minz, Omesh Kumar Bharti, Rupanjali Deka, Shivaprasad S. Goudar, Manjunath S. Somannavar, Sangappa M. Dhaded, Avinash Kavi, Deepthy M. Sadanandan, Archana Patel, Kunal Kurhe, Prabir Kumar Das, Vaishali Khedikar, Alka Dafare, Chittaranjan S. Yajnik, Urmila Deshmukh, Onkar Deshmukh, Sadhana Joshi, Girija Wagh, Sanjay Lalwani, Sanjay Gupte, Juhi Nema, Sunita Taneja

**Affiliations:** a Nuffield Department of Population Health, University of Oxford, Oxford, UK; b Department of Medical Statistics, London School of Hygiene & Tropical Medicine, London, UK; c PHFI-Centre for Developmental and Lifecourse Research, Indian Institute of Public Health Bengaluru, Public Health Foundation of India, Bengaluru, India; d Translational Health Science and Technology Institute, Faridabad, Haryana, India; e Society for Applied Studies, New Delhi, India; f Department of Biostatistics, St. John's Medical College, Bangalore, India; g Division of Nutrition, St. John's Research Institute, Bangalore, India; h Department of Obstetrics and Gynaecology, MediCiti Institute of Medical Sciences, Hyderabad, India; i Department of Population Medicine, College of Medicine, Qatar University, Doha, Qatar; j Gauhati Medical College, Guwahati, Assam, India; k J N Medical College, KLE Academy of Higher Education and Research, Belagavi, India; l Lata Medical Research Foundation, Nagpur, India; m Adjunct Faculty-Medical Research, DattaMeghe Institute of Higher Education and Research, Sawangi, India; n Diabetes Unit, KEM Hospital and Research Centre, Pune, India; o Mother and Child Health, ICMR Collaborating Centre of Excellence, Interactive Research School for Health Affairs, Bharati Vidyapeeth University, Pune, India; p Division of Reproductive, Child Health and Nutrition, Indian Council of Medical Research, New Delhi, India; q Global Health Section, Department of Public Health, University of Copenhagen, Denmark

**Keywords:** Stillbirth, Anaemia, Pregnancy, India

## Abstract

**Background:**

India contributed to 17.3% of the global stillbirth burden in 2019 and anaemia, an important risk factor for stillbirth, affects nearly half of pregnant women in the country. To understand the relationship between maternal anaemia and stillbirth, we analysed a diverse multicohort dataset from the Stillbirth Pooled Indian Cohort Consortium (ICMR-SPIC) from India.

**Methods:**

This was a secondary analysis of the ICMR-SPIC dataset, which includes individual-level data from eight observational studies and two randomised controlled trials conducted across ten states in India. Most cohorts recruited participants between 2010 and 2023, except for the Pune Maternal Nutrition Study (PMNS), which recruited participants between 1994 and 1996. A total of 214,709 singleton pregnant women with known birth outcomes and haemoglobin measurements during pregnancy were included. Unadjusted and adjusted risk ratios for the association between maternal anaemia and stillbirth were estimated using modified Poisson regression for each cohort and pooled using a random-effects meta-analysis. Kaplan–Meier survival curves were estimated for foetal survival beyond 28 weeks of gestation across categories of anaemia severity.

**Findings:**

The median maternal age of the women was 23 years (IQR 21–25). There were 108,982 (52%) women with moderate to severe anaemia (haemoglobin [Hb] <10 g/dL) and 3595 (1.7%) stillbirths. We found that moderate to severe anaemia at any point during pregnancy was associated with a higher risk of stillbirth after 28 weeks of gestation (aRR 1.27, 95% CI 1.07–1.51). The risk ratio for severe anaemia (<7 g/dL) was 3.12 (95% CI 2.46–3.97), and for moderate anaemia it was 1.20 (95% CI 1.02–1.40) at any gestational age, compared to women with no or mild anaemia. We found that stillbirths occurred around the 29th week of gestation among pregnant women with severe anaemia, compared to around 31 weeks of gestation among other women.

**Interpretation:**

These findings highlight that maternal anaemia is an important and potentially modifiable risk factor for stillbirth, with severe anaemia associated with early occurrence of stillbirth. Policies aimed at reducing stillbirths should prioritise comprehensive anaemia prevention and treatment strategies among pregnant women.

**Funding:**

Indian Council of Medical Research.

**Research in context**

**Evidence before this study**

We searched PubMed, Ovid, Embase, and medrxiv for studies reporting association between maternal anaemia and stillbirth. We used search terms synonymous with maternal anaemia, haemoglobin, and stillbirth, but restricted the search to studies between January 2000 and April 2026, although some studies have shown association between maternal anaemia and the risk of stillbirth in India, there is limited generalisable evidence originating from diverse setting across varying severity of anaemia and different trimesters.

**Added value of this study**

Our study leverages a multi-cohort dataset of pregnant women with known birth outcomes from the Stillbirth Pooled Indian Cohort (ICMR-SPIC consortium), to understand the association between severity of maternal anaemia and the risk of stillbirth. The dataset consists of nearly 220,000 pregnant women from ten cohorts across the country representing different states, communities, and urban and rural regions. This study leverages different statistical and regression methods to account for potential confounders across all cohorts to provide a pooled risk estimate. We found that maternal anaemia (haemoglobin levels <10 g/dL) at any point in the pregnancy is associated with the increased risk of stillbirth after 28 weeks of gestation, in India compared to no/mild anaemia (≥ 10 g/dL). Women with mild anaemia did not have increased risk for stillbirth except during the 2nd trimester. Stillbirths occurred earlier in gestation among those with severe anaemia at a median of 29 weeks of gestation while it was 31 weeks for moderate/mild/no anaemia.

**Implications of all the available evidence**

This study strengthens evidence on association between maternal anaemia and stillbirth for India. Interventions that address the modifiable causes of anaemia at any stage of pregnancy, through both therapeutic and public health approaches may help reduce the risk of stillbirth, whereas genetic and other non-modifiable causes will require alternative strategies. In addition, pregnant women with severe anaemia should be closely monitored to mitigate the risk of stillbirth. Taken together, these findings underscore the need for comprehensive policies that target modifiable risk factors, including anaemia, as part of broader efforts to accelerate progress in reducing stillbirths in India.

## Introduction

Stillbirth is a major global health challenge.^1^ It can have substantial psychological and social impact, including adverse effects on parents’ mental health, reduced economic productivity, and increased health care costs.^2^ Commendable progress has been made in reducing the global median stillbirth rate from 22.6 per 1000 births in 2000 to 14.3 per 1000 births in 2023, bringing it closer to the global target of 12 per 1000.^1^^,^^3^ However, Low- and middle-income countries (LMICs) continue to bear a disproportionately high burden of stillbirth.^4^^,^^5^ India contributed to 17.3% of the global stillbirth burden in 2019.^4^ Although multiple policies have been implemented across India to improve access to quality health care and maternal and newborn health outcomes, stillbirth burden in the country remains high.^6^

Anaemia, an important risk factor for stillbirth, affects higher proportion of women and girls in LMICs.^7^^,^^8^ Anaemia has multiple causes, including micronutrient deficiencies, infection, and genetic factors.^7^ Iron deficiency is the leading cause of anaemia globally.^5^ According to the National Family Health Survey—5 (2019–21), 52.2% of pregnant women in India were reported to be affected by anaemia.^9^ Within the Indian context, only a few studies have examined the association between maternal anaemia and stillbirth.^10^, ^11^, ^12^, ^13^ Existing studies have accounted for limited number of potential confounding factors in the exposure-outcome pathway, such as gestational diabetes (GDM), hypertensive disorders of pregnancy (HDP), and thyroid disorders. In addition, few studies have investigated the trimester-specific association between anaemia and stillbirth. There is also a paucity of generalisable evidence from India, which is needed to inform context-specific policies and interventions.

We sought to understand the role of maternal anaemia in influencing the risk of stillbirth. We aimed to examine the association between moderate-to-severe maternal anaemia at any point during pregnancy, as well as during each trimester, and stillbirth after 28 weeks of gestation while accounting for potential confounders. We also compared the timing of stillbirth occurrence among pregnant women with moderate and severe anaemia relative to those with no or mild anaemia in India.

## Methods

This was a secondary analysis of the Stillbirth Pooled India Cohort Consortium (ICMR-SPIC) dataset. The dataset compiles individual-level data from eight observational study cohorts and two randomised controlled trials (RCTs) conducted across ten states in India, with details described in the dataset profile paper.^14^ Supplementary Figure S1 presents the geographic distribution of the cohorts and their sample sizes. Across all cohorts, pregnant women were recruited antenatally, with the majority of participants enrolled from rural settings.^14^ Most cohorts recruited participants between 2010 and 2023, except for PMNS, which recruited participants between 1994 and 1996. Each cohort was designed to address a different research question. For example, the MaatHRI cohort aimed to understand the safety of induction and augmentation of labour among pregnant women with anaemia, whereas the CalPreg cohort examined the effects of calcium supplementation on pre-eclampsia and eclampsia. Multiple studies have been published using data from these cohorts. Of the two RCTs, both intervention and control arms from CalPreg were included, whereas only the control arm from WINGS was included. The intervention arm of WINGS was excluded because the intervention comprised health, nutrition, psychosocial care and support, and water, sanitation, and hygiene (WASH) measures during the preconception and pregnancy period which could possibly bias the analysis.^15^ Data from all cohorts were cleaned and harmonised by a team constituted by the Indian Council of Medical Research (ICMR).

All singleton pregnant women with at least one haemoglobin (Hb) measurement during pregnancy and known gestational age (GA) at birth and foetal outcomes were included. Pregnant women with missing information on these variables were excluded. To ensure consistency across cohorts, all variables were harmonised within the ICMR-SPIC dataset through standardisation of variable names and coding schemes. The variables included in the analysis are described in Supplementary Table S1.

We defined stillbirth in accordance with WHO guidance and the definition used in India, as foetal death at or beyond 196 days (28^+0^ completed weeks) of gestational. Maternal anaemia was defined using WHO Haemoglobin cut-offs for pregnancy: <7.0 g/dL for severe anaemia and 7.0–9.9 g/dL for moderate anaemia. Haemoglobin values ≥ 10.0 g/dL were classified as no or mild anaemia, consistent with previous studies.^16^^,^^17^ Moderate and severe anaemia categories were combined into a single category because the number of stillbirths in the severe anaemia category was fewer than 5 in some cohorts (Table 1). For the trimester-wise analysis, WHO-recommended haemoglobin cut-offs for each trimester were applied, as detailed in Supplementary Table S2.^18^ For women with two or more haemoglobin measurements during pregnancy, the lowest recorded value was used.

**Table 1:** Number of stillbirths and live births in each cohort by categories of anaemia.

Cohort	No/Mild anaemia		Moderate anaemia		Severe anaemia	
	Live birth (%)	Stillbirth (%)	Live birth (%)	Stillbirth (%)	Live birth (%)	Stillbirth (%)
TOTAL	101,725 (98.5)	1504 (1.5)	106,813 (98.2)	1959 (1.8)	2576 (95.1)	132 (4.9)
Cal Preg	8515 (98.2)	158 (1.8)	1664 (97.3)	47 (2.7)	24 (96.0)	1 (4.0)
Garbh-Ini	412 (97.6)	10 (2.4)	6070 (98.1)	120 (1.9)	122 (97.6)	3 (2.4)
Life	536 (98.9)	6 (1.1)	185 (96.4)	7 (3.6)	9 (90.0)	1 (10.0)
MAASTHI	2345 (99.7)	8 (0.3)	486 (99.2)	4 (0.8)	7 (87.5)	1 (12.5)
MaatHRI	924 (98.7)	12 (1.3)	6894 (98.3)	116 (1.7)	1721 (95.2)	87 (4.8)
MNHR B	48,830 (98.5)	746 (1.5)	49,952 (98.3)	854 (1.7)	488 (95.1)	25 (4.9)
MNHR N	37,989 (98.6)	544 (1.4)	40,863 (98.1)	804 (1.9)	165 (95.1)	12 (4.9)
PMNS	566 (99.3)	4 (0.7)	175 (99.4)	1 (0.6)	8 (88.9)	1 (11.1)
ReVamp	822 (99.6)	3 (0.4)	277 (99.3)	2 (0.7)	3 (100)	0 (0.0)
Wings	786 (98.4)	13 (1.6)	247 (98.4)	4 (1.6)	29 (96.7)	1 (3.3)

We developed a Directed Acyclic Graph (DAG) (Supplementary Figure S2) based on the conceptual framework provided in Supplementary Figure S3, to identify potential confounders and effect modifiers in the association between maternal anaemia and stillbirth. Variables were selected based on empirical evidence and the DAG was used to guide the analysis. Variables available within individual cohort were included in the cohort-specific pooled analyses, whereas only variables available across all cohorts were used for the rest of the analysis.

### Statistical analysis

We computed summary statistics such as, means with standard deviations, or medians with interquartile ranges for continuous variables, and frequencies with percentages for categorical variables.

As the outcome (stillbirth) was binary, the risk ratio was chosen as the measure of effect for this study. To calculate the risk ratio, we used modified Poisson regression with robust standard errors.^19^ Risk ratios were chosen over odds ratios because the latter can overestimate the relative magnitude of effect if the prevalence of the outcome in the study population is not rare, and they can be interpreted more easily for policy and planning purposes.^19^ Robust standard errors are required as binary outcomes do not follow the Poisson distribution, which is assumed by the standard model.^19^, ^20^, ^21^

In each cohort, we built three analytic models:

• Model 0: Unadjusted
• Model 1: Adjusted for confounders that were available across all cohorts (maternal age at the time of pregnancy, education in years, BMI, and hypertensive disorders of pregnancy)
• Model 2: Variables included in Model 1 with further adjustments for cohort-specific confounders, which included maternal risk factors such as gestational diabetes mellitus (GDM), thyroid disorders, parity, ante-natal care visits, and location (a list of cohort-specific variables can be found in Supplementary Table S3)

To explore potential effect modification, an interaction term for the location of residence (rural/urban) in the exposure-outcome pathway was tested in the analysis. This was conducted in the MaatHRI cohort data, since it was a mixed cohort where individual-level location data were available. Most other cohorts were homogeneous in terms of the location of residence of the participants.

For each cohort, three models were fitted based on the type of adjustment (described above). Cal Preg, MAASTHI, PMNS, ReVamp, and Wings were not included in this analysis as they had five or fewer stillbirths in the moderate-severe anaemia categories. Estimates for each model (Models 0, 1, and 2) were pooled across all cohorts using random-effects meta-analysis to account for inherent heterogeneity across cohorts. Cohorts with less than five stillbirths in the moderate-severe anaemia group were not included in the meta-analysis. Heterogeneity was assessed using the I^2^ statistic. The results of the meta-analysis were illustrated using a forest-plot. Sub-group analyses using location (urban, rural, mixed), size of study (N > 7500 categorised as large), and year of recruitment (2010 onwards or 2015 onwards) were also conducted to explore possible sources of heterogeneity in the pooled estimates.

Mixed-effects modified Poisson regression was also conducted on the combined dataset, similar to an individual participant data (IPD) meta-analysis. We performed four additional analyses in the combined dataset

1 Pregnant women were grouped into separate severe anaemia and moderate anaemia categories to estimate the risk ratio in each of these groups compared with the no/mild anaemia category.
2 Pregnant women were stratified into trimester-wise groups (for whom this data was available) based on the gestational age at which their haemoglobin levels were measured. These groups were then used to assess the trimester-wise associations between haemoglobin levels and anaemia categories and stillbirth.
3 We then examined the association between change in haemoglobin levels between the first and second, and second and third trimesters and risk of stillbirth, using modified Poisson regression in a subset of women with haemoglobin measurements across all three trimesters.

Additionally, we conducted an unadjusted time-to-event analysis using the Kaplan–Meier method. All birth outcomes other than stillbirth were censored. The log-rank test was used to compare survival curves across anaemia severity categories to assess whether each severity category differed statistically from the baseline category of no/mild anaemia. We checked the proportional hazards assumption and found it to be violated (Global Schoenfeld test p-value <0.0001) because of the anaemia categories and BMI. Therefore, we accounted for the violation by including a log-transformed interaction term between time (gestational age), anaemia categories, and BMI to compute the time-adjusted Cox proportional hazard ratios for the moderate-severe anaemia category compared with no/mild anaemia. The model adjusted for confounders that were available across all cohorts (maternal age at the time of pregnancy, education in years, BMI, and hypertensive disorders of pregnancy).

Missing data across all cohorts were assumed to be missing-at-random. Most cohort studies were not designed to investigate stillbirth as the primary outcome; therefore, missingness was unlikely to be related to the outcome. The main analysis was based on complete cases. We also conducted a sensitivity analysis using multiple imputation by chained equations (MICE) to explore the robustness of the main analysis to missingness.

The analyses were conducted using RStudio version 4·4·3 and STATA version 18·5. All effect estimates were considered statistically significant at a two-tailed p-value of less than 0.05.

### Ethics statement

All cohorts had requisite ethical approvals in India. The requirement for consent was waived since this study is a secondary analysis of the anonymised dataset compiled by ICMR. Details of the Ethics approval along with number can be found in Supplementary Document S1.

### Role of the funding source

The funders had no role in the study design, data collection, data analysis, interpretation, writing of the manuscript.

## Results

The analysis included 229,617 pregnant women recruited across all ten cohorts with 214,709 participants meeting the inclusion criteria, and the multivariable models included 209,742 complete cases (Supplementary Figure S4). The cohort-wise number of participants included in the analysis is presented in Supplementary Table S5.

Almost half (48%) of included women were categorised as having no/mild anaemia and 108,982 women (52%) were categorised as either moderate (106,355, 50.7%) or severe (2,627, 1.3%) anaemia. The median maternal age at pregnancy was 23 years (IQR 21–25), the median years of education was 6 years (IQR 6–12), and the median BMI was 19.9 kg/m^2^ (IQR 18.1–21.9).

There were 3595 (1.7%) stillbirths in the study sample, of which 2091 (58.2%) stillbirths were among women with moderate-severe anaemia. The stillbirth rate ranged from 0.45% (5 stillbirths, 1107 total births) in the ReVamp cohort to 2.2% (217 stillbirths; 9566 total births) in the MaatHRI cohort, and the total stillbirth rate for the harmonised dataset was 1.7%. The proportion of stillbirth was 1.5%, 1.8%, and 4.9% in the no/mild, moderate, and severe anaemia categories, respectively. The distribution of stillbirths in each cohort is shown in Table 1.

We found no evidence of interaction between anaemia and stillbirth by rural/urban residence of the pregnant woman in the MaatHRI cohort (interaction p-value 0.91), we modelled residence location as a potential confounder in subsequent analyses as majority of the participants (89%) were from rural setting.

Since the number of stillbirths were small, the moderate and severe anaemia categories were merged. In all cohorts, except Garbh-Ini, pregnant women with moderate-severe anaemia had a higher risk of stillbirth compared to pregnant women with no/mild anaemia (Table 2). However, the adjusted risk ratio for stillbirth was statistically significant only in Cal Preg and MNHR Nagpur cohorts. Since the risk ratios remained comparable across adjustment types, results from the model adjusting for common variables (mentioned above) were chosen as the main model and are shown in Table 2. The risk of stillbirth increased by 34% in MNHR Nagpur cohort and 58% in CalPreg cohort among pregnant women with moderate-severe anaemia. Results from the sensitivity analysis using multiple imputations were similar to the results from the complete case analysis and are provided in Supplementary Table S6.

**Table 2:** Unadjusted and adjusted risk ratios for showing the association between maternal anaemia and stillbirth (moderate-severe anaemia versus no-mild anaemia).

Cohort	Complete cases^a^	Anaemia category	Unadjusted Risk ratio (95% CI)	p-value	Adjustment 1^b^ Risk ratio (95% CI)	p-value	Adjustment 2^c^ Risk ratio (95% CI)	p-value
Cal Preg	10,408	No-Mild	1 (ref)		1 (ref)		1 (ref)	
		Mod-Sev	1.52 [1.10–2.09]	0.010	1.58 [1.16–2.18]	0.005	1.62 [1.18–2.24]	0.003
Garbh-Ini	5971	No-Mild	1 (ref)		1 (ref)		1 (ref)	
		Mod-Sev	0.91 [0.43–1.93]	0.80	0.89 [0.42–1.90]	0.77	0.92 [0.43–2.00]	0.39
Life	426	No-Mild	1 (ref)		1 (ref)		1 (ref)	
		Mod-Sev	1.94 [0.33–11.47]	0.47	2.274 [0.60–8.60]	0.23	1.53 [0.30–7.80]	0.83
MaatHRI	9334	No-Mild	1 (ref)		1 (ref)		1 (ref)	
		Mod-Sev	1.67 [0.94–2.99]	0.08	1.49 [0.83–2.67]	0.30	1.50 [0.83–2.71]	0.30
MNHR Belgavi	98,403	No-Mild	1 (ref)		1 (ref)		1 (ref)	
		Mod-Sev	1.13 [1.03–1.25]	0.012	1.08 [0.98–1.19]	0.13	1.10 [0.99–1.12]	0.07
MNHR Nagpur	80,197	No-Mild	1 (ref)		1 (ref)		1 (ref)	
		Mod-Sev	1.38 [1.24–1.54]	<0.0001	1.34 [1.20–1.50]	<0.0001	1.35 [1.21–1.51]	<0.0001

a As described in the methods section, only complete cases were included in this analysis, that is, participants who had all the available data on all the variables included in the model.

b Variables adjusted for maternal age, maternal education in years, maternal body mass index, and hypertensive disorders of pregnancy.

c Variables adjusted for cohort-specific confounders mentioned in Supplementary Table S3.

**Fig. 1:**
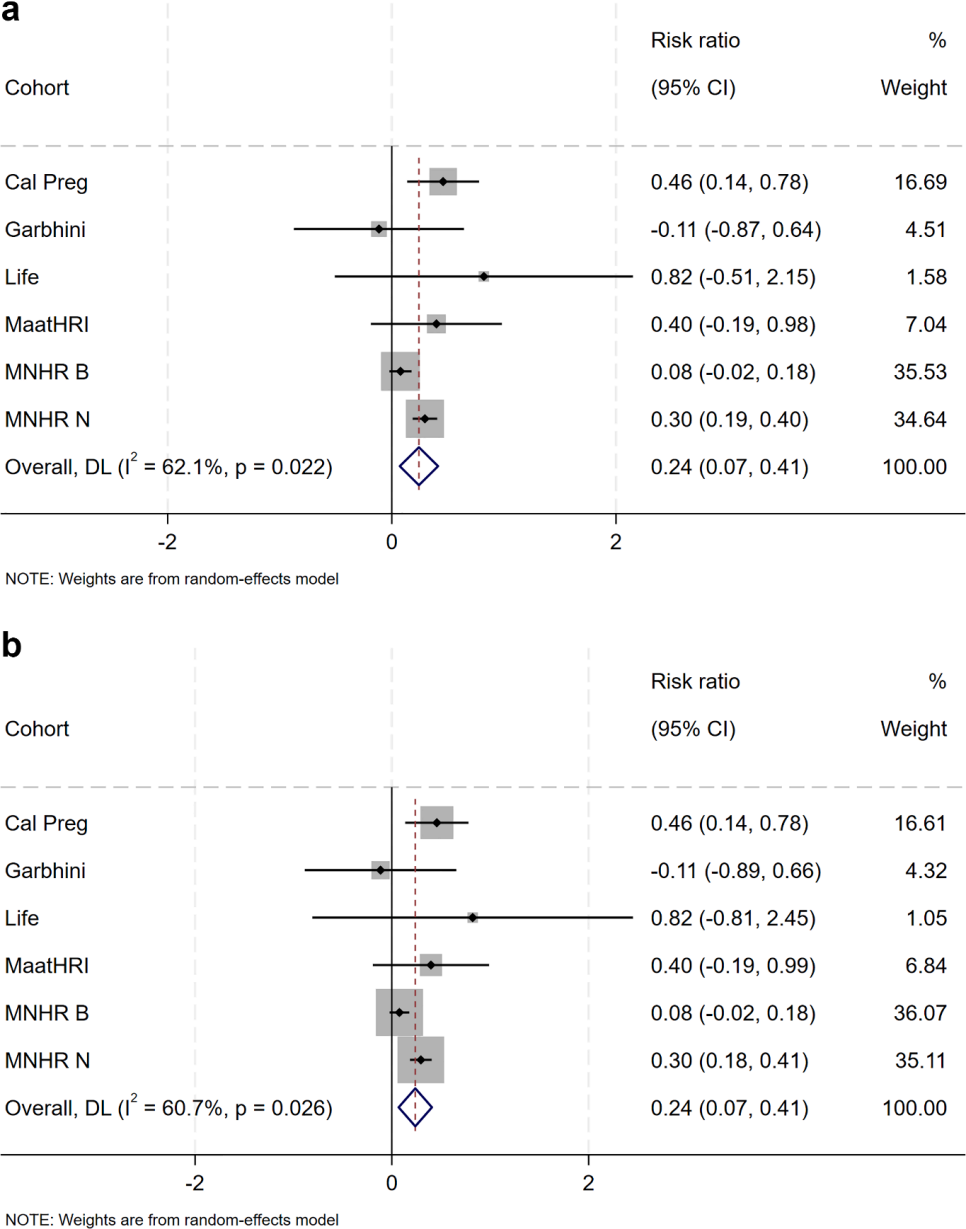
The pooled effects of moderate-severe anaemia on stillbirth in the ICMR-SPIC consortium dataset. The pooled risk ratios for six cohorts (Cal Preg, Garbhini, Life, MaatHRI, MNHR Belgaavi, MNHR Nagpur) included in the analysis. (a) Shows the pooled risk ratio adjusted for maternal age, education, body mass index (BMI), and hypertensive disorders of pregnancy (HDP). (b) Shows the pooled risk ratio adjusted for maternal age, education, BMI, HDP, and cohort-specific variables detailed in Supplementary Table S3. CI = Confidence Interval.

The pooled unadjusted risk ratio for stillbirth among moderate-severe anaemia was 1.30 (95% CI 1.12–1.50, p-value 0.001, I^2^ = 52.0%). The forest plot for the unadjusted risk ratio can be found in Supplementary Figure S5. In our main model, the pooled adjusted risk ratio using common confounders was 1.27 (95% CI 1.07–1.51, p-value 0.006, I^2^ = 62.1%), and adjusted ratio using cohort-specific confounders was 1.28 (95% CI 1.09–1.51, p-value 0.003, I^2^ = 57.8%) (Fig. 1).

In the subgroup analysis, I^2^ for the urban and mixed groups were negligible across all three pooled effect estimates but greater than 70% for the rural group, indicating that the rural group contributed to most of the heterogeneity amongst the cohorts. The forest plots for the subgroup analysis by location, year of participant recruitment, and study size can be found in Supplementary Figure S6.

The analysis using the combined data (like an IPD meta-analysis) showed that there was a 24% higher unadjusted and 20% higher adjusted risk of stillbirth among pregnant women with moderate anaemia category. For severe anaemia, unadjusted and adjusted risk ratio were 3.44 and 3.12 respectively (Table 3).

**Table 3:** Unadjusted and adjusted risk ratios showing the association between moderate and severe anaemia categories and stillbirth, compared to no-mild anaemia.

Anaemia severity	Unadjusted		Adjusted^a^	
	Risk Ratio (95% CI)	p-value^b^	Risk Ratio (95% CI)	p-value^b^
No-Mild Anaemia (n = 103,229)	1 (ref)		1 (ref)	
Moderate Anaemia (n = 108,772)	1.24 [1.08–1.43]	0.003	1.20 [1.02–1.40]	0.026
Severe Anaemia (n = 2708)	3.44 [2.83–4.18]	<0.0001	3.12 [2.46–3.97]	<0.0001

a Adjusted for common confounding variables—maternal age, maternal education, HDP, and BMI.

b Global p-value = <0.001.

The number of stillbirths and live births by anaemia category for each trimester is given in Supplementary Table S7. Trimester wise analysis showed significant risk of stillbirth among women with moderate and severe anaemia (Table 4). The risk of stillbirth among women with mild anaemia was not statistically significant, except in the second trimester.

**Table 4:** Adjusted risk ratios showing trimester-wise associations between maternal haemoglobin levels and moderate-severe anaemia and stillbirth.

Trimester	Anaemia category^a^ (n)	Adjusted Risk Ratio^b^ (95% CI)	p-value
First trimester (n = 69,622)	No (13,876)	1 (ref)	
	Mild (24,341)	1.03 [0.95–1.12]	0.51
	Moderate (31,138)	1.21 [1.04–1.41]	0.014
	Severe (267)	2.42 [0.86–6.81]	0.09
Second trimester (n = 60,558)	No (18,761)	1 (ref)	
	Mild (23,390)	1.23 [1.11–1.37]	<0.0001
	Moderate (18,055)	1.31 [1,06–1.62]	0.013
	Severe (352)	2.14 [1.16–3.94]	0.014
Third trimester (n = 120,780)	No (26,527)	1 (ref)	
	Mild (45,652)	0.94 [0.81–1.09]	0.42
	Moderate (47,026)	1.38 [0.98–1.94]	0.07
	Severe (1,575)	4.47 [3.22–6.22]	<0.0001

a Cut-offs for trimester-wise anaemia groups are given in Supplementary Table S2.

b Adjusted for common confounding.

Further, there were 4,240 women across cohorts who had at least one haemoglobin measurement in each trimester. We found no association between change in haemoglobin levels between first and second, and second and third trimesters, and the risk of stillbirth amongst all 4,240 women (Supplementary Table S8).

A total of 214,709 complete cases of pregnant women were included in time to event analysis. There were 3,595 stillbirths in this sample. Of these, 132 (3.6%) stillbirths were in the severe anaemia category, 1,959 (54.4%) in the moderate anaemia, and 1504 (42%) in the no/mild anaemia category. The mean time to childbirth was 38.78 weeks (standard deviation (SD) 2.38) in the no/mild anaemia category, 38.66 weeks (SD 2.48) in the moderate anaemia category, and 38.13 weeks (SD 2.43) in the severe anaemia category.

**Fig. 2:**
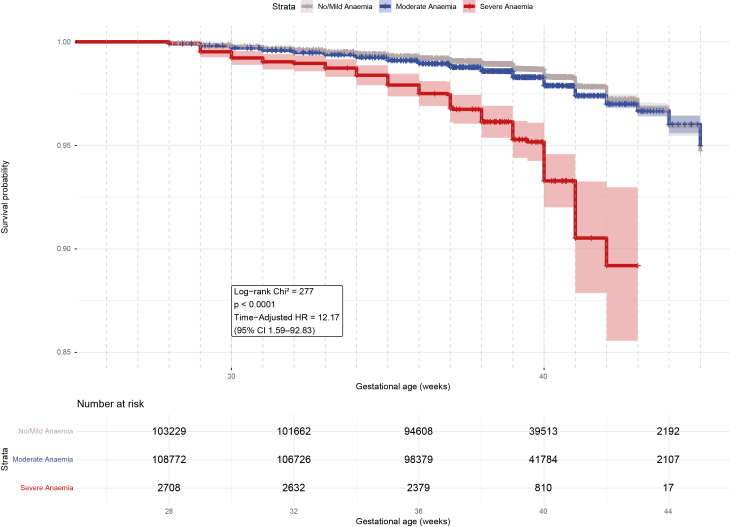
Kaplan–Meier curve comparing the survival probabilities for foetus born to pregnant women with moderate and severe anaemia and those with no/mild anaemia. The survival probability of the foetus stratified by severity of maternal anaemia, across increasing gestational age (in weeks). The proportional hazard assumption was not met when tested using the Schoenfeld test, thus, adjusted hazard ratio was calculated using an interaction term between time, anaemia categories, and BMI for the moderate-severe anaemia category compared with no/mild anaemia.

The survival curve for severe anaemia was steeper than moderate and no/mild anaemia, indicating a lower cumulative survival probability consistent with the findings from the combined dataset analyses. The adjusted hazard ratio, while accounting for the time-varying effects of anaemia categories and BMI, was 12.17 (95% CI 1.59–92.83, p-value 0.016) for the moderate-severe anaemia category when compared to the no/mild anaemia category. The 95% CI is wide, indicating a less precise estimate. The log-rank test χ^2^ was 277 with a p-value <0.0001, indicating that the curves for the moderate and severe anaemia categories were different from the no/mild anaemia category, and this difference was statistically significant (Fig. 2).

## Discussion

This secondary analysis showed that, when data from all cohorts were harmonised as a single group, women with moderate-to-severe anaemia at any time in the pregnancy had a 27% higher risk of stillbirth after 28 weeks of gestation. When anaemia was examined by severity, we found both severe and moderate anaemia were associated with a higher risk of stillbirth compared with no/mild anaemia. This association was observed consistently across all three trimesters of pregnancy. Furthermore, a reduction in the survival probability was observed among babies born to women with severe anaemia, with almost half occurring earlier in pregnancy at around 29th week of gestation compared with other categories of anaemia. Although mild anaemia was prevalent with large proportion of pregnant women affected in our dataset, the risk of stillbirth among women with mild anaemia was similar to that among women with no anaemia in the first and third trimesters but was higher than no-anaemia in the second trimester (Hb cut off 9.5–10.4 g/dL). This difference may reflect the trimester-specific haemoglobin cut-offs used to define mild anaemia.^18^

Studies from India have reported similar observations. Altijani et al. (2018) studied the relationship between maternal anaemia and stillbirth across India using the Annual Health Survey data for 886,505 women. They reported 35% higher adjusted odds of stillbirth among women with any anaemia.^10^ However, they observed a stillbirth rate of 10 per 1000 births compared to 16.7 per 1000 births in the ICMR-SPIC dataset. Similar results were seen in a study conducted by Parks et al., in 2019, in India and Pakistan, where the authors also found a higher risk of stillbirth among pregnant women with severe anaemia compared with pregnant women with no anaemia, but no statistically significant relationship for other categories of anaemia.^11^ Parks et al. included two cohorts (MNHR Belgaavi and MNHR Nagpur) from the ICMR-SPIC consortium in addition to other cohorts from Pakistan. The inclusion of additional cohorts could explain the differences in the study results, and thus, further strengthening the value of our observations in providing a comprehensive picture. Population-level evidence from a geospatial analysis of stillbirth rates (SBR) using HMIS data from India identified districts with high prevalence of anaemia and high stillbirth, further support our observations.^22^ Dandona et al. reported that anaemia was common among women who experiences stillbirths and highlighted multiple pregnancy complications.^23^ Further, Mittal et al. found that in India, women with anaemia were less likely to have stillbirths if they had four or more antenatal check-ups, suggesting that better antenatal care may reduce stillbirth risk in pregnant women with anaemia.^24^

Our findings were also consistent with global three meta-analyses.^25^, ^26^, ^27^ Young et al. reported a pooled 43% higher odds of stillbirth amongst women with low haemoglobin (<11 g/dL) at any point in pregnancy.^25^ They also found significantly higher odds of stillbirth during the first and second trimester among pregnant women with anaemia.^25^ Similarly, Kasa et al. reported a higher adjusted odds of stillbirth among pregnant women with anaemia across 12 studies from Sub-Saharan Africa.^26^ Wang et al. reported comparable findings, they pooled 10 studies from LMICs and observed a 26% higher risk of stillbirths among pregnant women with anaemia.^27^ None of the above studies analysed severity and trimester specific risk of association of anaemia with stillbirth.

A secondary analysis of nearly 19 million pregnant women from China using data from the Hospital Quality Monitoring System showed that the adjusted odds of stillbirth was 86% higher among those with severe anaemia but had lower odds of 21% and 41% among those with moderate and mild anaemia.^28^ The lower prevalence of anaemia and stillbirth in the above study could be attributable to the observed variation in risk difference. Other studies from Kenya, Tanzania, and China observed no association between maternal anaemia and stillbirth.^29^, ^30^, ^31^ These variations could be attributed to differences in the definitions of exposure and outcome, smaller samples, and variation in potential confounders included for analysis in the study.

Currently, there are two theories to explain possible pathways for anaemia and stillbirth, Maternal anaemia leading to embryonic anaemia may cause oxidative stress in the foetus, leading to poor outcomes. This pathway is likely mediated by low birthweight for gestational age due to weakened placental circulation, causing foetal growth restriction, which in severe cases may lead to stillbirth.^32^^,^^33^ Maternal anaemia could cause cardiovascular defects in the offspring, which could result in stillbirth in some foetuses.^32^^,^^34^ These pathways were not directly examined in this study, since this line of investigation was beyond the scope, but future research could delineate these mechanisms to inform preventative measures.

The survival curve (Fig. 2) showed that stillbirths start to occur earlier (a median of 29th week of gestation) among those with severe anaemia compared to a median of 31 weeks among those with moderate and no/mild anaemia. This suggests the need for early intervention, including active monitoring and increasing haemoglobin levels in pregnant women with severe anaemia.

This study has some limitations. The term definition for anaemia included all types, nutritional deficiency, genetic factors, and infection-based anaemia. Information on the root cause of anaemia could have helped improve existing knowledge on the risk variation by type of anaemia. Each cohort included in this study was designed to answer a different research question, resulting in inconsistent data on potential confounders; however, the exposure and outcome were consistently described. Analysing each cohort individually and then pooling the results helped overcome this challenge to some extent, but residual confounding cannot be ruled out. Some cohorts had very few stillbirths in the moderate-severe anaemia category, which resulted in them being excluded from the analysis. Different statistical techniques were used to overcome this problem, including combining all cohorts to represent all regions of the country and prevent loss of statistical power.

This secondary analysis provides a comprehensive assessment of the association between maternal anaemia and stillbirth in India. The findings from this study have important implications for policy and practice. We found that both moderate and severe anaemia at any time during pregnancy were associated with a higher risk of stillbirth after 28 weeks of gestation. Women with severe anaemia had reduced foetal survival probability and is associated with a higher risk for stillbirth at around 29th week of gestation, emphasising the need for early identification and timely management. Taken together, these findings suggest that efforts to reduce stillbirth should prioritise the prevention, detection, and management of moderate and severe anaemia during pregnancy. Treatments and interventions that aim to improve modifiable causes of anaemia at any point in the pregnancy could contribute to reducing stillbirth risk in India.

## Contributors

PG designed the study protocol as part of her DPhil project, under the supervision of MN and CO. RM, convener of the ICMR-SPIC consortium, facilitated access to and provided the dataset used in this study. DM contributed critical feedback and guidance during the study design phase. RC, PD, NW, KB, GRB, SSC, SSG, AP, CSY, SJ, and MN were either the Principal Investigators or part of the investigating team for the individual cohort studies included in the ICMR-SPIC consortium and were responsible for data collection, data verification, maintenance, and contribution to the consortium dataset. Ay, RM, JMR, RT, and RC were responsible for compiling and cleaning the complete dataset. Statistical analyses were conducted by PG under the supervision of MN and CO as part of her DPhil project. The final results were reviewed and approved by all authors. The manuscript was drafted by PG under the supervision of MN and CO. All authors reviewed and approved the final version of the manuscript.

## Data sharing statement

Data can be obtained by contacting Reema Mukherjee from ICMR, joint senior author on the paper. The R code for this study is available at https://github.com/PrernaGopal/Anaemia-Stillbirth-ICMR-SPIC-analysis.

## Editor note

The Lancet Group takes a neutral position with respect to territorial claims in published maps and institutional affiliations.

## Declaration of interests

PG is a DPhil student at the Nuffield Department of Population Health, University of Oxford and is funded by a Departmental scholarship. MN has received funding from an MRC Transition award and a UKRI Developmental Pathway Gap Fund. MN has a start package grant from the Novo Nordisk Foundation in her current role as Professor of Global Health at University of Copenhagen, and is also supported by University of Oxford and Copenhagen.

## Appendix A Supplementary data

Supplementary Material

Collab Authors
