# Supplementary material for: Risk of stillbirth among pregnant women with anaemia in India: secondary analysis of multiple cohorts from the ICMR-SPIC consortium
Source: Lancet Reg Health Southeast Asia. 2026 Jul 31;52:100825. doi: 10.1016/j.lansea.2026.100825 (PMC13453048; doi:10.1016/j.lansea.2026.100825)
Supplement: Supplementary file 1 [file mmc1.docx]

**Risk of stillbirth among pregnant women with anaemia in India: secondary analysis of multiple cohorts from the ICMR-SPIC consortium**

**Authors: Prerna Gopal, Charles Opondo, Debarati Mukherjee, Ayushi, Rukman Manapurath, John Michael Raj, Ramachandran Thiruvengadam, Ranadip Chowdhury,** **Pratibha Dwarkanath, Nitya Wadhwa, Kalpana Basany, Giridhar Rathnaiah Babu, Saswati Sanyal Choudhury, Shivaprasad S Goudar, Archana Patel, Chittaranjan S Yajnik, Sadhana Joshi, Sunita Taneja, Reema Mukherjee*, Manisha Nair*, and ICMR-SPIC consortium^#^**

# **Supplementary Files**

Contents

[**Supplementary Files**	1](#_Toc230184264)

[**Supplementary Files:**	3](#_Toc230184265)

[Supplementary Table 1: Covariates	3](#_Toc230184266)

[Supplementary Table 2: WHO cut-offs for anaemia across different trimesters	4](#_Toc230184267)

[Supplementary Table 3: Cohort specific variables using in Adjustment 2	5](#_Toc230184268)

[Supplementary Table 4: Descriptive Summary by cohort	6](#_Toc230184269)

[Supplementary Table 5: Cases included in the final analytical sample	10](#_Toc230184270)

[Supplementary table 6: Results from the multiple imputation sensitivity analysis	10](#_Toc230184271)

[Supplementary table 7: Number of stillbirth and livebirths by anaemia category for each trimester	11](#_Toc230184272)

[Supplementary table 8: Results from analysis for women with haemoglobin reading across all three trimesters	11](#_Toc230184273)

[**Supplementary Figures**	12](#_Toc230184274)

[Supplementary Figure 1: Map of India depicting the states where the cohort studies are based along with sample size	12](#_Toc230184275)

[Supplementary Figure 2: Directed Acyclic Graph showing the relationship between maternal anaemia and stillbirth created using DAGGITY^1^	13](#_Toc230184276)

[Supplementary Figure 3: Conceptual framework used to design DAG for the analysis	14](#_Toc230184277)

[Supplementary Figure 4: Study sample based on the inclusion criteria	15](#_Toc230184278)

[Supplementary Figure 5: Pooled risk ratio from unadjusted effect estimates for each cohort	16](#_Toc230184279)

[Supplementary Figure 6: Subgroup analysis to test heterogeneity	17](#_Toc230184280)

[**Supplementary Documents**	26](#_Toc230184281)

[Supplementary Document 1: Ethics Approval for ICMR-SPIC cohort	26](#_Toc230184282)

# **Supplementary Files:**

## Supplementary Table 1: Covariates

| S.No. | Variable | Data type | Codes | Cohorts where variable is available |
| --- | --- | --- | --- | --- |
| Exposure and outcome | | | | |
| 1 | Stillbirth during index pregnancy (outcome variable) | Binary | Stillbirth was coded as ‘1’, no stillbirth as ‘0’, Data not collected was coded as ‘NA’ | All cohorts |
| 2 | Maternal anaemia category for trimester-wise analysis | Categorical | Women with severe anaemia (< 7 g/dl) were coded as ‘2’, those with moderate anaemia (7 to 9.9 g/dl) were coded as ‘1’, women with Hb value ≥10 g/dl are coded as ‘0’ | All cohorts |
| 3 | Maternal anaemia for pooled analysis | Categorical | Women with Hb value less than 10 g/dL were coded as ‘1’, women with Hb value greater than and equal to 10 g/dl are coded as ‘0’ | All cohorts |
| Confounders | | | | |
| 1 | Number of ANC visits | Categorical | 0-1 ANC visits was coded as ‘0’, 2 ANC visits as ‘1’, 3 ANCs as ‘2’, and >=4 ANC visits as ‘3’ | All cohorts except MAASTHI |
| 2 | Body mass index of pregnant woman during pregnancy | Continuous | As per BMI ratio. We considered the lowest weight at any point in the pregnancy to calculate the BMI. | All cohorts |
| 3 | Gestational diabetes | Categorical | Presence of GDM coded as ‘1’, no GDM as ‘0’, and data not collected was coded as ‘NA’ | All cohorts except CalPreg, MNHR B, MNHR N, and Wings |
| 4 | Hypertensive Disorders in Pregnancy | Categorical | Presence of preeclampsia, gestational hypertension, eclampsia, or a blood pressure reading of greater than 140/90    at any point in pregnancy were coded as ‘1’, otherwise ‘0’, Data not collected was coded as ‘NA’. This was based on the best practices definition by an expert panel. The standard definition of HDP includes chronic hypertension, which was not included in this study. | All cohorts except MNHR N |
| 5 | Maternal education in years | Continuous | In years | All cohorts |
| 6 | Location | Categorical | Women from urban locations were coded as ‘1’, women from rural locations as ‘0’, Data not collected is coded as ‘NA’ | Only MaatHRI cohort. Most other cohorts have a homogenous location except Garbh-Ini which is mixed but this variable is unavailable. |
| 7 | Maternal age | Continuous | Completed years | All cohorts |
| 8 | Parity Category | Ordinal | Nulliparous women were coded as ‘0’, Parity of 1 is coded as ‘1’, Parity of 2 as ‘2’, Parity of 3 as ‘3’, Parity 4 and above is coded as ‘4’, Data not collected was coded as ‘NA’ | All cohorts. In case of CalPreg, all women were nulliparous |
| 9 | Thyroid disorder | Categorical | Hypothyroidism was coded as ‘1’, hyperthyroidism was coded as ‘2’, Euthyroid as ‘0’, and Data not collected was coded as ‘NA’ | All cohorts except CalPreg, MNHR B, MNHR N, and PMNS |

## Supplementary Table 2: WHO cut-offs for anaemia across different trimesters

|  | First trimester  < 12 weeks | Second Trimester  13 – 27 weeks | Third Trimester  28 weeks to birth |
| --- | --- | --- | --- |
| No anaemia | >= 11 g/dL | >= 10·5 g/dL | >= 11 g/dL |
| Mild anaemia | 10 – 10·9 g/dL | 9·5 – 10·4 g/dL | 10 – 10·9 g/dL |
| Moderate anaemia | 7 – 9·9 g/dL | 7 – 9·4 g/dL | 7 – 9·9 g/dL |
| Severe anaemia | < 7 g/dL | < 7 g/dL | < 7 g/dL |

## Supplementary Table 3: Cohort specific variables using in Adjustment 2

| **Cohort** | **Cohort-specific variables** |
| --- | --- |
| Calpreg | Maternal age, Maternal education, BMI, HDP, ANC visits |
| Garbh-Ini | Maternal age, Maternal education, BMI, HDP |
| LiFE | Maternal age, Maternal education, BMI, ANC visits, HDP, GDM, thyroid disorder |
| MAASTHI | Maternal age, Maternal education, BMI, HDP, GDM, thyroid disorder |
| MaatHRI | Maternal age, Maternal education, BMI, HDP, ANC visits, location |
| MNHR Belgavi | Maternal age, Maternal education, BMI, HDP, Parity |
| MHNR Nagpur | Maternal age, Maternal education, BMI, HDP, Parity |
| Pune | Maternal age, Maternal education, BMI, HDP, ANC visits, GDM |
| ReVamp | Maternal age, Maternal education, BMI, ANC visits, HDP, GDM, parity |
| Wings | Maternal age, Maternal education, BMI, HDP, ANC visits, thyroid disorder, GDM |

## Supplementary Table 4: Descriptive Summary by cohort

| Cohort > | Total | CalPreg | Garbh-Ini | Life | MAASTHI | MaatHRI | MHNR-B | MNHR-N | PMNS | ReVamp | Wings |
| --- | --- | --- | --- | --- | --- | --- | --- | --- | --- | --- | --- |
| Sample size | N=214,709 | N=10,409 | N=6,737 | N=744 | N=2,851 | N=9,754 | N=100,895 | N=80,377 | N=755 | N=1,107 | N=1,080 |
| Continuous Variables |  |  |  |  |  |  |  |  |  |  |  |
| Maternal haemoglobin levels at any point in pregnancy (in g/dL) | 9·8 (9·2-10·3) | 10·8 (10·2-11·4) | 8·7 (8·2-9·0) | 10·8 (9·8-11·8) | 11·1 (10·3-11·8) | 8·3 (7·3-9·0) | 9·9 (9·2-10·3) | 9·8 (9·2-10·2) | 11·0 (10·0-11·7) | 10·6 (9·9-11·3) | 10·9 (9·9-11·9) |
| Gestational age at delivery (in weeks) | 39·0 (38·0-40·0) | 39·1 (38·0-40·0) | 39·0 (38·0-40·0) | 39·0 (38·0-40·0) | 39·0 (38·0-39·6) | 39·0 (38·0-40·0) | 39·0 (38·0-40·0) | 39·0 (38·0-40·0) | 39·0 (38·0-39·9) | 38·6 (37·9-39·4) | 39·0 (37·9-39·9) |
| Maternal BMI* | 19·9 (18·1-22·0) | 22·5 (19·6-26·1) | 20·2 (18·3-22·7) | 19·8 (18·1-22·8) | 24·7 (21·7-28·0) | 20·5 (18·7-22·4) | 20·0 (18·2-21·9) | 19·4 (17·8-21·3) | 18·8 (17·7-20·0) | 23·6 (20·7-26·7) | 22·1 (19·5-25·2) |
| Maternal height** | 152·2 (5·5) | 153·7 (5·5) | 153·0 (5·8) | 151·9 (5·8) | 154·1 (5·7) | 151·5 (6·1) | 152·2 (5·4) | 151·9 (5·5) | 151·9 (5·0) | 155·7 (6·2) | 152·1 (5·5) |
| Inter-pregnancy interval* | 26·0 (18·0-37·0) |  | 18·0 (8·0-36·0) | 15·0 (10·0-24·0) |  | 33·0 (20·0-51·0) | 28·0 (22·0-38·0) | 23·0 (15·0-34·0) |  | 33·0 (18·0-59·0) |  |
| Maternal Education  (in years)* | 6·0 (6·0-12·0) | 10·0 (10·0-12·0) | 10·0 (6·0-12·0) | 10·0 (7·0-10·0) | 10·0 (10·0-12·0) | 12·0 (5·0-12·0) | 6·0 (0·0-6·0) | 12·0 (10·0-12·0) | 7·0 (2·0-9·0) | 17·0 (12·0-17·0) | 12·0 (8·0-14·0) |
| Maternal Age  (in years)* | 23·0 (21·0-25·0) | 23·0 (21·0-26·0) | 23·0 (21·0-26·0) | 22·0 (20·0-23·0) | 24·0 (21·0-27·0) | 24·0 (21·0-28·0) | 23·0 (20·0-25·0) | 24·0 (22·0-25·0) | 21·0 (19·0-23·0) | 28·5 (25·3-31·7) | 24·0 (22·0-26·0) |
| Birth weight  (in grams)* | 2736·6 (441·1) | 2823·8 (457·5) | 2768·1 (511·7) | 2761·2 (494·3) | 2865·9 (467·5) | 2666·9 (468·7) | 2773·9 (423·1) | 2682·0 (436·2) | 2420·2 (763·0) | 2882·9 (469·0) | 2763·8 (479·3) |
|  |  |  |  |  |  |  |  |  |  |  |  |
| Categorical Variables | n (%) | n (%) | n (%) | n (%) | n (%) | n (%) | n (%) | n (%) | n (%) | n (%) | n (%) |
| Antenatal Check-ups |  |  |  |  |  |  |  |  |  |  |  |
| No ANC | 3,041 (1·4) | 78 (0·7) | 1,412 (21·0) | 7 (0·9) | NA | 612 (6·3) | 315 (0·3) | 563 (0·7) | 34 (4·5) | 0 (0·0) | 20 (1·9) |
| 1 ANC | 5,915 (2·8) | 239 (2·3) | 1,167 (17·3) | 14 (1·9) | 0 (0·0) | 1,283 (13·2) | 1,907 (1·9) | 970 (1·2) | 276 (36·6) | 10 (0·9) | 49 (4·5) |
| 2 ANC | 20,366 (9·5) | 480 (4·6) | 2,674 (39·7) | 43 (5·8) | 0 (0·0) | 3,463 (35·5) | 9,455 (9·4) | 3,689 (4·6) | 445 (58·9) | 45 (4·1) | 72 (6·7) |
| 3 or more ANCs | 88,303 (41·1) | 9,612 (92·3) | 1,484 (22·0) | 644 (86·6) | 0 (0·0) | 4,198 (43·0) | 33,087 (32·8) | 37,299 (46·4) | 0 (0·0) | 1,052 (95·0) | 927 (85·8) |
| Missing | 97,084 (45·2) | 0 (0·0) | 0 (0·0) | 36 (4·8) | 2,851 (100·0) | 198 (2·0) | 56,131 (55·6) | 37,856 (47·1) | 0 (0·0) | 0 (0·0) | 12 (1·1) |
| Alcohol Consumption |  |  |  |  |  |  |  |  |  |  |  |
| No | 21,341 (9·9) | 0 (0·0) | 6,714 (99·7) | 636 (85·5) | 2,518 (88·3) | 9,659 (99·0) | 0 (0·0) | 0 (0·0) | 755 (100·0) | 1,059 (95·7) | 0 (0·0) |
| Yes | 264 (0·1) | 0 (0·0) | 10 (0·1) | 108 (14·5) | 3 (0·1) | 95 (1·0) | 0 (0·0) | 0 (0·0) | 0 (0·0) | 48 (4·3) | 0 (0·0) |
| Missing | 193,104 (89·9) | 10,409 (100·0) | 13 (0·2) | 0 (0·0) | 330 (11·6) | 0 (0·0) | 100,895 (100·0) | 80,377 (100·0) | 0 (0·0) | 0 (0·0) | 1,080 (100·0) |
| Unclean fuel |  |  |  |  |  |  |  |  |  |  |  |
| No | 54,726 (25·5) | 10,323 (99·2) | 6,213 (92·2) | 534  (71·8) | 2,708 (95·0) | 0 (0·0) | 8,849 (8·8) | 23,970 (29·8) | 0 (0·0) | 1,049 (94·8) | 1,080 (100·0) |
| Yes | 39,188 (18·3) | 24 (0·2) | 133 (2·0) | 210  (28·2) | 0 (0·0) | 0 (0·0) | 26,153 (25·9) | 12,659 (15·7) | 0 (0·0) | 9 (0·8) | 0 (0·0) |
| Missing | 120,795 (56·3) | 62 (0·6) | 391 (5·8) | 0 (0·0) | 143 (5·0) | 9,754 (100·0) | 65,893 (65·3) | 43,748 (54·4) | 755 (100·0) | 49 (4·4) | 0 (0·0) |
| Gestational Diabetes |  |  |  |  |  |  |  |  |  |  |  |
| No | 15,647 (7·3) | 0 (0·0) | 1,293 (19·2) | 579  (77·8) | 2,440 (85·6) | 9,728 (99·7) | 0 (0·0) | 0 (0·0) | 667 (88·3) | 940 (84·9) | 0 (0·0) |
| Yes | 964 (0·4) | 0 (0·0) | 346 (5·1) | 12  (1·6) | 411 (14·4) | 26 (0·3) | 0 (0·0) | 0 (0·0) | 4 (0·5) | 165 (14·9) | 0 (0·0) |
| Missing | 198,098 (92·3) | 10,409 (100·0) | 5,098 (75·7) | 153 (20·6) | 0 (0·0) | 0 (0·0) | 100,895 (100·0) | 80,377 (100·0) | 84 (11·1) | 2 (0·2) | 1,080 (100·0) |
| Previous abortion |  |  |  |  |  |  |  |  |  |  |  |
| No | 8,849 (4·1) | 0 (0·0) | 2,268 (33·7) | 0 (0·0) | 2,297 (80·6) | 3,402 (34·9) | 0 (0·0) | 0 (0·0) | 681 (90·2) | 201 (18·2) | 0 (0·0) |
| Yes | 2,980 (1·4) | 0 (0·0) | 1,828 (27·1) | 104 (14·0) | 552 (19·4) | 6 (0·1) | 0 (0·0) | 0 (0·0) | 74 (9·8) | 416 (37·6) | 0 (0·0) |
| Missing | 202,880 (94·5) | 10,409 (100·0) | 2,641 (39·2) | 640 (86·0) | 2 (0·1) | 6,346 (65·1) | 100,895 (100·0) | 80,377 (100·0) | 0 (0·0) | 490 (44·3) | 1,080 (100·0) |
| Hypertensive disorders during pregnancy | | | |  |  |  |  |  |  |  |  |
| No | 206,109 (95.9) | 9952 (95.6) | 5384 (80.0) | 601 (80.8) | 2628 (92.8) | 8876 (91.0) | 97694 (96.4) | 78450 (97.6) | 740 (98.0) | 891 (80.4) | 893 (82.8) |
| Yes | 7,647  (4.096) | 457 (4.4) | 693 (10.0) | 140 (18.8) | 223 (7.80) | 878 (9.0) | 3042 (3.02) | 1888 (2.4) | 15 (2.0) | 216 (19.6) | 95 (8.7) |
| Missing | 953  (0.004) | 0 (0·0) | 660 (10.0) | 3 (0.004) | 0 (0·0) | 0 (0·0) | 159 (0.001) | 39 (0.00) | 0 (0·0) | 0 (0·0) | 92 (8.5) |
| Location |  |  |  |  |  |  |  |  |  |  |  |
| Rural | 190,339 (88·6) | 0 (0·0) | 0 (0·0) | 744 (100·0) | 0 (0·0) | 8,312 (85·2) | 100,895 (100·0) | 80,377 (100·0) | 755 (100·0) | 0 (0·0) | 0 (0·0) |
| Urban | 16,889 (7·9) | 10,409 (100·0) | 0 (0·0) | 0 (0·0) | 2,851 (100·0) | 1,442 (14·8) | 0 (0·0) | 0 (0·0) | 0 (0·0) | 1,107 (100·0) | 1,080 (100·0) |
| Mixed | 7,481 (3·5) | 0 (0·0) | 6,737 (100·0) | 0 (0·0) | 0 (0·0) | 0 (0·0) | 0 (0·0) | 0 (0·0) | 0 (0·0) | 0 (0·0) | 0 (0·0) |
| Parity |  |  |  |  |  |  |  |  |  |  |  |
| Nulliparous | 101,477 (47·3) | 10,409 (100·0) | 3,399 (50·5) | 50 (6·7) | 1,257 (44·1) | 6,543 (67·1) | 39,795 (39·4) | 38,810 (48·3) | 242 (32·1) | 674 (60·9) | 298 (27·6) |
| Parity = 1 | 76,734 (35·7) | 0 (0·0) | 2,208 (32·8) | 332 (44·6) | 1,304 (45·7) | 2,048 (21·0) | 36,790 (36·5) | 32,957 (41·0) | 261 (34·6) | 394 (35·6) | 440 (40·7) |
| Parity = 2 | 26,368 (12·3) | 0 (0·0) | 859 (12·8) | 129 (17·3) | 265 (9·3) | 745 (7·6) | 17,181 (17·0) | 6,770 (8·4) | 163 (21·6) | 37 (3·3) | 219 (20·3) |
| Parity = 3 | 6,858 (3·2) | 0 (0·0) | 211 (3·1) | 16 (2·2) | 21 (0·7) | 255 (2·6) | 4,858 (4·8) | 1,353 (1·7) | 59 (7·8) | 1 (0·1) | 84 (7·8) |
| Parity >= 4 | 2,672 (1·2) | 0 (0·0) | 60 (0·9) | 2 (0·3) | 2 (0·1) | 163 (1·7) | 1,918 (1·9) | 457 (0·6) | 30 (4·0) | 1 (0·1) | 39 (3·6) |
| Missing | 600 (0·3) | 0 (0·0) | 0 (0·0) | 215 (28·9) | 2 (0·1) | 0 (0·0) | 353 (0·3) | 30 (0·0) | 0 (0·0) | 0 (0·0) | 0 (0·0) |
| Passive Smoking |  |  |  |  |  |  |  |  |  |  |  |
| No | 50,019 (23·3) | 0 (0·0) | 5,479 (81·3) | 742 (99·7) | 1,906 (66·9) | 0 (0·0) | 26,927 (26·7) | 13,164 (16·4) | 755 (100·0) | 1,046 (94·5) | 0 (0·0) |
| Yes | 20,371 (9·5) | 0 (0·0) | 1,252 (18·6) | 0 (0·0) | 916 (32·1) | 0 (0·0) | 8,210 (8·1) | 9,932 (12·4) | 0 (0·0) | 61 (5·5) | 0 (0·0) |
| Missing | 144,319 (67·2) | 10,409 (100·0) | 6 (0·1) | 2 (0·3) | 29 (1·0) | 9,754 (100·0) | 65,758 (65·2) | 57,281 (71·3) | 0 (0·0) | 0 (0·0) | 1,080 (100·0) |
| Stillbirths |  |  |  |  |  |  |  |  |  |  |  |
| No | 211,114 (98·3) | 10,203 (98·0) | 6,604 (98·0) | 730 (98·1) | 2,838 (99·5) | 9,539 (97·8) | 99,270 (98·4) | 79,017 (98·3) | 749 (99·2) | 1,102 (99·5) | 1,062 (98·3) |
| Yes | 3,595 (1·7) | 206 (2·0) | 133 (2·0) | 14 (1·9) | 13 (0·5) | 215 (2·2) | 1,625 (1·6) | 1,360 (1·7) | 6 (0·8) | 5 (0·5) | 18 (1·7) |
| Thyroid Disorders |  |  |  |  |  |  |  |  |  |  |  |
| Euthyroid | 20,809 (9·7) | 0 (0·0) | 6,603 (98·0) | 576 (77·4) | 2,554 (89·6) | 9,699 (99·4) | 0 (0·0) | 0 (0·0) | 0 (0·0) | 709 (64·0) | 668 (61·9) |
| Hypothyroid | 1,009 (0·5) | 0 (0·0) | 84 (1·2) | 14 (1·9) | 196 (6·9) | 42 (0·4) | 0 (0·0) | 0 (0·0) | 0 (0·0) | 291 (26·3) | 382 (35·4) |
| Hyperthyroid | 78 (0·0) | 0 (0·0) | 6 (0·1) | 1 (0·1) | 0 (0·0) | 13 (0·1) | 0 (0·0) | 0 (0·0) | 0 (0·0) | 28 (2·5) | 30 (2·8) |
| Missing | 192,813 (89·8) | 10,409 (100·0) | 44 (0·7) | 153 (20·6) | 101 (3·5) | 0 (0·0) | 100,895 (100·0) | 80,377 (100·0) | 755 (100·0) | 79 (7·1) | 0 (0·0) |

Notes: *Median, Inter-quartile range **Mean, standard deviation

## Supplementary Table 5: Cases included in the final analytical sample

| Cohort | Original sample | Final Sample | % Excluded |
| --- | --- | --- | --- |
| Calpreg | 10,544 | 10,409 | 1% |
| Garbh-Ini | 6874 | 6,737 | 2% |
| LiFE | 1,269 | 744 | 41% |
| MAASTHI | 3,280 | 2,851 | 13% |
| MaatHRI | 10,109 | 9,754 | 4% |
| MNHR Belgavi | 111,620 | 100,895 | 10% |
| MHNR Nagpur | 82,207 | 80,377 | 2% |
| Pune | 770 | 755 | 2% |
| ReVamp | 1,745 | 1,107 | 37% |
| Wings | 1,099 | 1,080 | 2% |
| Total sample size | **229,617** | **214,709** | **6%** |

## Supplementary table 6: Results from the multiple imputation sensitivity analysis

| **Cohort** | **Risk Ratio with multiple imputation (95% CI)** | **p-value** |
| --- | --- | --- |
| CalPreg | 1 (ref) |  |
|  | 1.64  [1.19-2.27] | 0.002 |
| Garbhini | 1 (ref) |  |
|  | 0.85  [0.45-1.62] | 0.622 |
| Life | 1 (ref) |  |
|  | 2.38  [0.79-7.20] | 0.125 |
| MaatHRI | 1 (ref) |  |
|  | 1.66  [0.92-3.01] | 0.095 |
| MNHR-Belgaavi | 1 (ref) |  |
|  | 1.16  [1.002-1.22] | 0.044 |
| MNHR-Nagpur | 1 (ref) |  |
|  | 1.35  [1.21-1.51] | <0.001 |

## Supplementary table 7: Number of stillbirth and livebirths by anaemia category for each trimester

|  | First trimester  (n = 69,622) | | Second trimester  (n = 60,558) | | Third trimester  (n =120,780) | |
| --- | --- | --- | --- | --- | --- | --- |
|  | **Live birth** | **Still birth** | **Live birth** | **Still birth** | **Live birth** | **Still birth** |
| No Anaemia | 13,666 | 210 | 18,453 | 308 | 26,212 | 315 |
| Mild Anaemia | 23,978 | 363 | 22,880 | 510 | 45,166 | 486 |
| Moderate Anaemia | 30,575 | 563 | 17,645 | 410 | 46,265 | 761 |
| Severe Anaemia | 256 | 11 | 340 | 12 | 1483 | 92 |

## Supplementary table 8: Results from analysis for women with haemoglobin reading across all three trimesters

| Centile group | N | Median (IQR) | Risk ratio  [95% CI] | p-value |
| --- | --- | --- | --- | --- |
| *Change in Hb measurement in first to second trimester^2^* | 4,240 | 0·000  (-0.60-0.50) | 1.13  [0.85-1.49] | 0.389 |
| *Change in Hb measurement in second to third trimester^3^* | 4,240 | 0·00  (-0.80-0.60) | 1.00  [0.79-1.25] | 0.967 |

Note: (1) Adjusted for maternal age, maternal education, HDP, and BMI (2) Change was calculated as: Hb in second trimester – Hb in first trimester (3) Change was calculated as : Hb in third trimester – Hb in second trimester

# **Supplementary Figures**

## Supplementary Figure 1: Map of India depicting the states where the cohort studies are based along with sample size

CalPreg

10,544

Garbh-Ini

6,874

Wings

1,099

LIFE

1,269

Maasthi

3,280

MNHR N, MNHR B, PMNS

194,597

MaatHRI

10,109

## Supplementary Figure 2: Directed Acyclic Graph showing the relationship between maternal anaemia and stillbirth created using DAGGITY^1^

## Supplementary Figure 3: Conceptual framework used to design DAG for the analysis

## Supplementary Figure 4: Study sample based on the inclusion criteria

**Distribution of anaemia in the sample:**

No/Mild anaemia: 48.0%

Moderate anaemia: 50.7%

Severe anaemia: 1.3%

Participants recruited across all 10 cohorts

**229,617**

Participants excluded

**14,908**

Total pregnant women in the study

**214,709**

Total sample in analysis using categorical exposure (6 cohorts)

**204,813**

Total sample in analysis using the combined dataset (10 cohorts)

**214,709**

Total complete cases in analysis using the combined dataset (10 cohorts)

**209,742**

## Supplementary Figure 5: Pooled risk ratio from unadjusted effect estimates for each cohort

## Supplementary Figure 6: Subgroup analysis to test heterogeneity

**Association between maternal anaemia category during pregnancy and stillbirth**

1.1. **Sub-group analysis by Location for pooled effect estimate using unadjusted risk ratios**

1.2. **Sub-group analysis by year of recruitment for pooled effect estimate using unadjusted risk ratios**

1.3. **Sub-group analysis by study size for pooled effect estimate using unadjusted risk ratios**

**2.1 Sub-group analysis by location for pooled effect estimate using adjusted risk ratios (adjusted for common variables: maternal age, maternal education, BMI, and HDP)**

2.2 **Sub-group analysis by year of recruitment for pooled effect estimate using adjusted risk ratios (adjusted for common variables: maternal age, maternal education, BMI, and HDP)**

2.3 **Sub-group analysis by study size for pooled effect estimate using adjusted risk ratios (adjusted for common variables: maternal age, maternal education, BMI, and HDP)**

**3.1	Sub-group analysis by Location for pooled effect estimate using adjusted risk ratios (adjusted for cohort-specific variables – see Supplementary Table 3)**

**3.2. Sub-group analysis by year of recruitment for pooled effect estimate using adjusted risk ratios (adjusted for cohort-specific variables – see Supplementary Table 3)**

**3.3 Sub-group analysis by study size for pooled effect estimate using adjusted risk ratios (adjusted for cohort-specific variables – see Supplementary Table 3)**

# **Supplementary Documents**

## Supplementary Document 1: Ethics Approval for ICMR-SPIC cohort

As documented in the Cohort profile paper, all cohorts included in this consortium have obtained requisite ethics approval to collect and share data.^2^ Please see an excerpt from the profile paper below:

*“The IEC ID numbers are mentioned below as follows: Cohort (in alphabetical order); Name of the Ethics Committee; and Reference ID.*

*(1) CalPreg cohort; Institutional Ethical Review Board, St. John's Medical College, Bangalore 560034; Ref. No. 197/2017 (14 September, 2017; original); amendments and extensions: 1 January, 2018; 24 July, 2018; 9 September, 2018; 30 April, 2020; 5 June, 2020; 12 November, 2020.*

*(2) GARBHINI cohort; Institutional Ethics Committee, Translational Health Science and Technology Institute, Faridabad, India, and Institutional Ethics Committee, Gurugram Civil Hospital, Haryana, India; THS/1.8.1/(71), dated 26 August 2019, GHG/IEC letter, dated 3 September 2019.*

*(3) LIFE cohort; MediCiti Ethics Committee, MediCiti Institute of Medical Sciences Campus, Ghanpur, Medchal Mandal, Telangana 501401; (1) Initial study approved by IEC vide its letters dated 26 May 2008; 7 June 2008; (2) Children follow- up for 6–7 years approved by Ethics Committee vide its letter dated 31 January 2017; (3) Children follow- up for 8–16 years approved by IEC vide its letter dated 4 October 2018.*

*(4) MAASTHI cohort; Institutional Ethics Committee at Bangalore campus of Indian Institute of Public Health - Hyderabad; Indian Institute of Public Health - Hyderabad Technical Review Committee Institutional Ethics Committee - 091- 2015, dated 13 November 2015. Renewed annually until 2020.*

*(5) MNHR- Belagavi cohort; Institutional Ethics Committee of KLE Academy of Higher Education and Research, Belagavi; Ref. No.: MDC/IECHSR/2007- 08/D- 6989- 7002, dated 19 January 2008. (6) MNHR Nagpur cohort; Research Ethics Review Committee, Lata Medical Research Foundation, Nagpur, India; LMRF/SGCT/RPC01/01/04/2009.*

*(7) PMNS cohort; King Edward Memorial Hospital Research Center, Pune, Maharashtra, India; KEMHRC/LFG/Dir.Offi/Adhoc/2175- A, dated 22 March 2024.*

*(8) REVAMP cohort; Bharati Vidyapeeth Medical College Institutional Ethical Committee; IEC/2015/37, dated 3 October 2015.*

*(9) WINGS cohort; Ethics Review Committee of the Society for Applied Studies, New Delhi, and Vardhman Medical College and Safdarjung Hospital, New Delhi, and WHO, Geneva; SAS/ERC/LG/2017 dated 3 June 2017, IEC/SJH/VMMC/PROJECT- 2017/694, ERC.0002934.*

*(10) MaatHRI cohort (Institution, Reference number and date of approval): Gauhati Medical College & Hospital: No. MC/190/2007/Pt- I/126, dated 18 January 2018. Mahatma Gandhi Institute of Medical Sciences: No. MGIMS/IEC/OBGY/118/2017, dated 15 December 2017. Institute of Medical Sciences, Banaras Hindu University: No. Dean/2018/EC/290, dated 2 January 2018. Nazareth Hospital: Ref. NH/CMO/IEC/ COMMUNICATIONS/18- 01, dated 2 January 2018. Emmanuel Hospital Association, New Delhi: No. 167, dated 19 January 2018. Dr Rajendra Prasad Government Medical College (Dr RPGMC), Kangra at Tanda, Himachal Pradesh, India: No. HFW- H DRPGMC/Ethics/2020/034, dated 25 June 2020. Oxford Tropical Research Ethics Committee (OxTREC): OxTREC Ref. 7- 18, dated 19 April 2018. Government of India’s Health Ministry’s Screening Committee, the Indian Council of Medical Research, New Delhi: No. 2018- 0152, dated 1 May 2018.”*

Reference:

1.	Textor J, van der Zander B, Gilthorpe MS, Liskiewicz M, Ellison GT. Robust causal inference using directed acyclic graphs: the R package 'dagitty'. *Int J Epidemiol* 2016; **45**(6): 1887-94.

2.	ICMR-SPICConsortium, Mukherjee R. Cohort profile of the ICMR-Stillbirth Pooled India Cohort (ICMR-SPIC): estimating prevalence, analysing risk factors and developing prediction models for stillbirths in India. *BMJ Open* 2025; **15**(8): e099401.
