# Supplementary material for: Risk of stillbirth among pregnant women with anaemia in India: secondary analysis of multiple cohorts from the ICMR-SPIC consortium
Source: Lancet Reg Health Southeast Asia. 2026 Jul 31;52:100825. doi: 10.1016/j.lansea.2026.100825 (PMC13453048; doi:10.1016/j.lansea.2026.100825)
Supplement: Supplementary file 2 [file mmc2.docx]

**#ICMR-SPIC Consortium Details:**

| **Cohort**  **(alphabetical order)** | **First Name** | **Last Name** | **Affiliation(s)** |
| --- | --- | --- | --- |
| Calcium in Pregnancy  (CalPreg) | Pratibha | Dwarkanath | Associate Professor, Division of Nutrition, St. John's Research Institute, Bangalore |
|  | John Michael | Raj | Lecturer, Department of Biostatistics, St. John's Medical College, Bangalore |
|  | Anura V | Kurpad | Professor, Department of Physiology, St. John's Medical College, Bangalore |
|  | Tinku | Thomas | Prof and Head, Department of Biostatistics, St. John's Medical College, Bangalore |
| Interdisciplinary Group for Advanced Research on Birth Outcomes - DBT India Initiative  (GARBHINI) | Rahul | Jain | Project Research Scientist-I, Translational Health Science and Technology Institute, Faridabad, India |
|  | Nitya | Wadhwa | Senior Professor, Translational Health Science and Technology Institute, Faridabad, India |
|  | Shinjini | Bhatnagar | Distinguished Professor, Translational Health Science and Technology Institute, Faridabad, India |
| Longitudinal Indian Family hEalth Pilot Study  (LiFE) | Kalpana | Basany | Professor and Head, Department of Obstetrics and Gynaecology,MediCiti Institute of Medical Sciences,Hyderabad |
|  | Shailendra | Dandge | Professor and Head,Department of Pharmacology,MediCiti Institute of Medical Sciences, SHARE INDIA,Hyderabad, India |
|  | Catherine L | Haggerty | Professor, Department of Epidemiology, University of Pittsburgh School of Public Health, USA |
|  | Late Dr Cleareann | H Bunker | Associate Professor Emerita, Department of Epidemiology, School of Public Health, University of Pittsburgh, USA |
|  | PS | Reddy | Professor of Medicine, University of Pittsburgh, USA |
| Maternal Antecedents of Adiposity and Studying Hyperglycemia  (MAASTHI) | Giridhara R | Babu | Professor, Department of Population Medicine, College of Medicine, Qatar University, Doha, Qatar. |
|  | Deepa | R | Research Scientist, PHFI-Centre for Developmental and Lifecourse Research, and Senior Lecturer, Indian Institute of Public Health-Bengaluru, Public Health Foundation of India |
|  | Suganya | Rajendran | Project Technical Assistant, PHFI-Centre for Developmental and Lifecourse Research, Indian Institute of Public Health-Bengaluru, Public Health Foundation of India, Bengaluru |
|  | Yamuna | Ana | Research Associate, Indian Institute of Public Health-Bengaluru, Public Health Foundation of India, Bengaluru |
| Maternal and Child Health Translational Research Initiative  (MaatHRI) | Manisha | Nair | Principal Investigator and Project Lead, MaatHRI project  Professor of Epidemiology & Global Health  Nuffield Department of Population Health, University of Oxford, UK  Professor of Global Health, Department of Public Health, Global Health Section, University of Copenhagen, Denmark |
|  | Saswati Sanyal | Choudhury | Professor of Obstetrics and Gynaecology, Guwahati Medical College, Guwahati, Assam |
|  | Farzana | Zahir | Assistant Professor of Obstetrics and Gynaecology, Department of Obstetrics and Gynaecology, Assam Medical College, Dibrugarh, Assam, India |
|  | Carolin | Solomi V | Consultant Obstetrician and Gynecologist, Department of Obstetrics and Gynaecology, Makunda Christian Leprosy and General Hospital, Karimganj, Assam, India |
|  | Ashok | Verma | Professor of Obstetrics and Gynaecology, Department of Obstetrics and Gynaecology, Dr Rajendra Prasad Government Medical College, Tanda, Himachal Pradesh, India |
|  | Sereesha | Rao | Obstetrician and Gynaecologist, Department of Obstetrics and Gynaecology, Silchar Medical College and Hospital, Silchar, Assam, India |
|  | Gitanjali | Deka | Associate Professor of Obstetrics and Gynaecology, Department of Obstetrics and Gynaecology, Gauhati Medical College and Hospital, Guwahati, Assam, India |
|  | Pranabika | Mahanta | Assistant Professor of Obstetrics and Gynaecology, Department of Obstetrics and Gynaecology, Tezpur Medical College, Tezpur, Assam, India |
|  | Swapna | Kakoty | Associate Professor of Community Medicine, Department of Obstetrics and Gynaecology, Jorhat Medical College and Hospital, Jorhat, Assam, India |
|  | Robin | Medhi | Professor and Head of Department of Obstetrics and Gynaecology, Department of Obstetrics and Gynaecology, Fakhruddin Ali Ahmed Medical College and Hospital, Barpeta, Assam, India |
|  | Shakuntala | Chhabra | Director and Professor of Obstetrics and Gynaecology, Department of Obstetrics and Gynaecology, Mahatma Gandhi Institute of Medical Sciences, Sevagram, Maharashtra, India |
|  | Anjali | Rani | Associate Professor of Obstetrics and Gynaecology, Department of Obstetrics and Gynaecology, Banaras Hindu University Institute of Medical Sciences, Varanasi, Uttar Pradesh, India |
|  | Amrit | Bora | Senior Medical Officer, Department of Obstetrics and Gynaecology, Sonapur District Hospital, Guwahati, Assam, India |
|  | Indrani | Roy | Consultant and Head of Department of Obstetrics and Gynaecology, Department of Obstetrics and Gynaecology, Nazareth Hospital, Shillong, Meghalaya, India |
|  | Bina | Minz | Consultant Obstetrician and Gynaecologist, Department of Obstetrics and Gynaecology, Sewa Bhawan Hospital Society, Basna, Chattisgarh, India |
|  | Omesh Kumar | Bharti | State Epidemiologist, Department of Health & Family Welfare, State Institute of Health and Family Welfare, Government of Himachal Pradesh, Shimla, Himachal Pradesh, India |
|  | Rupanjali | Deka | Project Manager, MaatHRI Project, Srimanta Sankaradeva University of Health Sciences, Guwahati, Assam, India |
| Maternal Newborn Health Registry  (MNHR)-Belagavi | Shivaprasad S | Goudar | Professor of Physiology, J N Medical College, and Director-Research, KLE Academy of Higher Education and Research, Belagavi, India |
|  | Manjunath S | Somannavar | Professor of Biochemistry, J N Medical College, KLE Academy of Higher Education and Research, Belagavi, India |
|  | Sangappa M | Dhaded | Professor and Head, Department of Neonatology, J N Medical College, KLE Academy of Higher Education and Research, Belagavi, India |
|  | Avinash | Kavi | Associate Professor of Community Medicine, J N Medical College, KLE Academy of Higher Education and Research, Belagavi, India |
|  | Deepthy M | Sadanandan | Research Scientist and Biostatistician, JNMC Women's and Children's Health Research Unit, J N Medical College, KLE Academy of Higher Education and Research, Belagavi, India |
| Maternal Newborn Health Registry  (MNHR)Nagpur | Archana | Patel | Program Director, Lata Medical Research Foundation, Nagpur, India  Adjunct Faculty-Medical Research, Datta Meghe Institute of Higher Education and Research, Sawangi, India |
|  | Kunal | Kurhe | Coordinator, Lata Medical Research Foundation, Nagpur, India |
|  | Prabir Kumar | Das | Country Coordinator, Lata Medical Research Foundation, Nagpur, India |
|  | Vaishali | Khedikar | Country Coordinator, Lata Medical Research Foundation, Nagpur, India |
|  | Alka | Dafare | Statistician, Lata Medical Research Foundation, Nagpur, India |
| Pune Maternal Nutrition Study  (PMNS) | Chittaranjan S | Yajnik | Director, Diabetes Unit, KEM Hospital and Research Centre, Pune, India |
|  | Urmila | Deshmukh | Consultant Pediatrician and Neonatologist, KEM Hospital and Research Centre, Pune, India |
|  | Onkar | Deshmukh | Statistician, KEM Hospital and Research Centre, Pune, India |
| Research Exploring Various Aspects and Mechanisms in Preeclampsia  (REVAMP) | Sadhana | Joshi | Professor and Head, Mother and Child Health, ICMR Collaborating Centre of Excellence, Interactive Research School for Health Affairs, Bharati Vidyapeeth University, Pune, India |
|  | Girija | Wagh | Professor and Head, Department of Obstetrics and Gynaecology, Bharati Hospital and Research Centre, Pune, India |
|  | Sanjay | Lalwani | Medical Director, Professor and Head, Dept of Paediatrics, Bharati Hospital and Research Centre, Pune, India |
|  | Sanjay | Gupte | Director, Gupte Hospital and Research Centre, Pune, India |
|  | Juhi | Nema | Assistant Professor, Mother and Child Health, ICMR Collaborating Centre of Excellence, Interactive Research School for Health Affairs, Bharati Vidyapeeth University, Pune, India |
| Women and Infants Integrated Interventions for Growth Study  (WINGS) | Sunita | Taneja | Senior Scientist, Society for Applied Studies, New Delhi, India |
|  | Neeta | Dhabhai | Senior Clinical Researcher, Society for Applied Studies, New Delhi, India |
|  | Nita | Bhandari | Senior Scientist and Director, Society for Applied Studies, New Delhi, India |
